# Post kala-azar dermal leishmaniasis burden at the village level in selected high visceral leishmaniasis endemic upazilas in Bangladesh

**DOI:** 10.1016/j.ijid.2024.107213

**Published:** 2024-10

**Authors:** Debashis Ghosh, Soumik Kha Sagar, Md Rasel Uddin, Md Utba Rashid, Shomik Maruf, Rupen Nath, Md Nazmul Islam, MM Aktaruzzaman, Abu Nayeem Mohammad Sohel, Megha Raj Banjara, Axel Kroeger, Abraham Aseffa, Dinesh Mondal

**Affiliations:** 1 Nutrition Research Division (NRD), International Centre for Diarrhoeal Disease Research, Bangladesh (ICDDR,B), Dhaka, Bangladesh; 2 Department of Epidemiology and Biostatistics, Arnold School of Public Health, University of South Carolina, Columbia, South Carolina, USA; 3 Communicable Disease Control (CDC), Directorate General of Health Services (DGHS), Mohakhali, Dhaka, Bangladesh; 4 UNICEF/UNDP/World Bank/World Health Organization Special Programme for Research and Training in Tropical Diseases (TDR), World Health Organization, Geneva, Switzerland; 5 Central Department of Microbiology, Tribhuvan University, Kirtipur, Kathmandu, Nepal; 6 Centre for Medicine and Society/Institute for Infection Prevention, University Medical Centre, Freiburg, Germany

**Keywords:** Post kala-azar dermal leishmaniasis, Prevalence, Knowledge, Stigma, Bangladesh

## Abstract

**Objectives:**

As post kala-azar Dermal Leishmaniasis (PKDL) threatens the success of the Visceral Leishmaniasis (VL) elimination initiative, we aimed to investigate the PKDL burden, including an active search for PKDL in leprosy-negative skin lesion cases. We also investigated their health-seeking behavior and perceived level of stigma.

**Methods:**

This was a cross-sectional survey among inhabitants in the VL-endemic villages of the five most VL-endemic upazilas. VL experts trained medical officers in Upazila Health Complexes (UHCs) and leprosy facilities in PKDL management. Frontline workers conducted house-to-house surveys, referring PKDL suspects to designated centers. Data analysis involved Epi Info version 7 and IBM SPSS Statistics 25.

**Results:**

Among 472,435 screened individuals, 4022 had past VL (0.85 %). Among the screened population, 82 were PKDL suspects, and 62 PKDL cases were confirmed. The overall PKDL burden was 1.3 (95 % CI: 1.0-1.7) in the 10,000 population in the endemic villages. Male predominance and macular form of PKDL were observed. Thirty-nine PKDL patients perceived stigma of different levels. Only 27 of 62 (44 %) had received PKDL treatment. Medicine's unavailability and side effects were a major reason behind treatment interruption. Active screening among 137 leprosy-negative PKDL suspects yielded 10 (7.3 %) PKDL cases.

**Conclusion:**

The existence of PKDL cases in the VL endemic areas is a concern as those are inter-epidemic reservoirs. As per the WHO roadmap, the PKDL burden must be reduced by 70 % and 100 %, respectively, by 2026 and 2030. NKEP can take the current burden of 1.3 per 10,000 people in VL endemic villages as a baseline. Integrating active case detection for PKDL in leprosy hospitals and screening centers is feasible and worth deploying nationwide.

## Highlights

• A total of 472,435 screened in 102 VL-endemic villages of five upazillas in Bangladesh.
• Sixty-two confirmed PKDL cases (75.6 %) from 82 suspected; 35 were new cases.
• Macular type was the most common PKDL form.
• A total of 44 % (27/62) confirmed PKDL cases sought medical attention for the lesion.
• Ten PKDL cases were confirmed from leprosy-negative patients.

## Introduction

Among the different manifestations of leishmaniasis, post kala-azar dermal leishmaniasis (PKDL) occurs in areas endemic to *Leishmania donovani*. Mostly, PKDL is a sequela of visceral leishmaniasis (VL) characterized by hypo-pigmented macular, papular, nodular, or polymorphic skin lesions. However, it can happen in individuals without a past history of VL but living in VL-endemic areas [[1], [2], [3]]. Skin lesions of PKDL are often confounded with other skin conditions, such as leprosy or vitiligo, causing difficulty in the differential diagnosis. PKDL is a chronic condition where patients serve as reservoirs for VL parasites, facilitating further transmission of VL in the communities [4]. Usually, PKDL patients do not seek medical care as they are clinically healthy. Therefore, active case search, diagnosis, and treatment of PKDL are essential to sustain the success of the VL elimination initiative in the Indian sub-continent.

The risk factors for PKDL are not fully understood. A study conducted in Bangladesh through an active case search for VL and PKDL cases showed that among 22,699 individuals surveyed, 813 (3.6 %) had VL at any time in the past, and 79 (0.35 %) had PKDL with eight additional PKDL patients without a history of VL [5]. Further, the cohort study in Bangladesh revealed that the development of PKDL and VL relapses (VLRs) are related to treatment regimens for VL. Sodium stibogluconate (SSG) and multi-dose liposomal amphotericin B (LAmB) resulted in less incidence of PKDL and VLRs compared to other treatment regimens [6]. Follow-up of VL patients treated with single dose AmBisome, AmBisome-miltefosine, and miltefosine-paromomycin in India between 2012 and 2014 revealed a PKDL incidence of 1.29, 1.45, and 2.65 per 1000-person months respectively. Children <12 years of age and females were in high-risk groups for PKDL [7]. A retrospective study conducted in 2010 in Nepal showed that among VL patients treated with sodium stibogluconate (SSG) between 2000 and 2009, 2.4 % had PKDL [8].

It is difficult to predict who will develop PKDL. Poor treatment compliance, young age (5-17 years), malnutrition, co-infection with HIV and treatment of HIV, and long-time environmental arsenic exposure may all be associated with the development of PKDL [9]. A recent xenodiagnosis study revealed that nodular and macular PKDL patients are infectious for sandflies, thus requiring prompt treatment [4]. PKDL is thus recognized as a threat to the VL elimination effort, and developing strategies for case finding, diagnosis, and treatment is one of the priority objectives of the Kala-azar Elimination Program. However, the actual burden of PKDL in Bangladesh is unknown. As per the WHO roadmap for eliminating neglected tropical diseases, the PKDL burden must be reduced by 70 % and 100 %, respectively, by 2026 and 2030 [10]. There is no routine active case search for PKDL in the national program, and baseline information about PKDL for the WHO roadmap is currently unavailable. Passive case detection does not reflect the real burden of PKDL in the VL communities, and an active case search in the community is a better way to determine the real burden of PKDL.

PKDL patients do not always feel sick and may not seek treatment. Such inappropriate treatment-seeking behavior of PKDL patients may allow the disease to stay chronic, increase its severity, and enhance its transmissibility [4]. Lack of knowledge and perceived stigma may cause patient delay in seeking treatment. Further, it is possible that PKDL cases without a past history of VL seek care in Leprosy hospitals since the clinical presentation of PKDL and leprosy is similar.

In this study, we aimed to determine the prevalence of PKDL in the VL endemic villages of highly VL endemic upazilas in Bangladesh, to explore feasibility and yield for active case research for PKDL in the leprosy hospitals and screening centers, and to identify the healthcare-seeking behavior and experience of stigma by PKDL patients in highly VL endemic upazilas in Bangladesh.

## Methodology

### Study design, sites, population, and sample size

**Figure 1:**
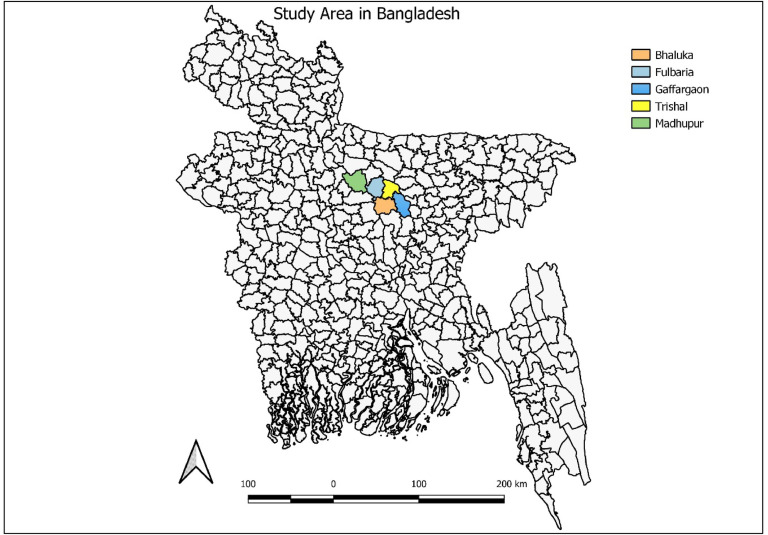
Five high-endemic areas for PKDL prevalence study.

This cross-sectional survey used a purposive sampling strategy and included all VL endemic villages (N = 102) of the five most highly endemic VL upazilas (∼ sub-districts) for the period 2018-2021 (Figure 1). We obtained the number of VL cases reported during the time frame from the National Kala-azar Elimination Program (NEKP). Then, we identified the upazillas with the highest number of VL cases. These were Fulbaria, Trishal, Bhaluka, and Gaffargoan under the Mymensingh district of Mymensingh Division, while Madhupur is a sub-district in the Tangail district of the Dhaka Division. The number of VL endemic villages was 33, 28, 17, 12, and 12, respectively, in Fulbaria, Trishal, Gaffargoan, Bhaluka and Madhupur. The total population in 102 villages was collected from the Population and Housing Census 2011 report by the Bangladesh Bureau of Statistics [11,12]. Then, the total population was projected using the following population projection equation.

$$P=P_{0}\;x\;\text{exp}\left(\text{rt}\right)$$

Where,

P = Total population
P_0_ = Starting population
r = Rate of growth of the sub-districts
t = Years since the census in 2011 (10 years)

The growth rate was 3.32, 1.23, 0.40, -3.43, and 1.17 for Bhaluka, Fulbaria, Gaffargaon, Madhupur, and Trishal, respectively. The final population in 102 VL endemic villages of this study was 472,435 as 201,717, 122,201, 70,853, 60,708, and 16,956 were from Fulbaria, Trishal, Gaffargoan, Bhaluka, and Madhupur respectively.

In addition, we deployed an integrated approach for PKDL case detection in the two leprosy hospitals (Jalchatra and Shambhugonj) and in the five leprosy screening centers based in the Upazila Health Complex(s) (UHCs) of the study areas. The Damien Foundation Bangladesh is involved in leprosy case detection and management in the Mymensingh and Dhaka Divisions. The Jalchatra Hospital covers three districts (Tangail, Jamalpur and Sherpur), and Shambugonj Hospital covers the Mymensingh, Netrokona and Kishoregonj districts. The screening centers at the UHCs cover only the upazila catchment areas. After successful coordination with Damien Foundation Bangladesh, we included the above-mentioned two leprosy hospitals and five leprosy screening centers in an active search for PKDL patients where individuals with skin lesions negative for leprosy underwent rK39 testing to detect PKDL. We carried out the study from May 2021 to June 2022.

### Training

VL experts trained medical officers, lab technicians, health inspectors, and assistant health inspectors from five UHCs on PKDL case detection, diagnosis, and management, and about the objectives and activities of the study in the Surya Kanta Kala-azar Research Centre (SKKRC) in Mymensingh. Health inspectors and assistant health inspectors guided the field research team in the field for village location and household identification. Lab technicians performed the rK39 rapid test at the field site during a house-to-house survey among suspected PKDL cases. rK39 positive probable PKDL cases were referred from the field by the research team to the nearest UHC, where the medical officer(s) confirmed the diagnosis and provided the treatment as per national guidelines for VL/PKDL case management [13]. The medical officers and laboratory technicians of the selected two leprosy hospitals and five leprosy centers were also trained on the differential diagnosis of PKDL. Laboratory technicians of the leprosy facilities performed the rk39 test among leprosy-negative patients, and rk39-positive cases were referred to the nearest UHCs for confirmation and subsequent treatment.

### Operational definitions

According to the National Kala-azar case management guideline of the National Kala-azar Elimination Program of Bangladesh (NKEP), an individual with a past history of VL or exposure to a VL endemic area with hypo-pigmented macule(s), papule(s) and or nodule(s) with skin sensation is a suspected case of PKDL. A suspected case of PKDL with a positive for the rK39 test is a probable case of PKDL. A confirmed PKDL case is defined by the demonstration of LD bodies/LD DNA in skin lesions. As per the guidelines, both probable and confirmed cases are eligible for treatment. Microscopic examination or molecular test of skin specimens are not available in upazila health complexes. So, a referred suspected PKDL case is eligible for treatment after confirmation as a probable case of PKDL by case history, physical examination, and repeat rK39 test. Herein, we have defined them as confirmed cases of PKDL.

### House-to-house survey

House-to-house survey activity was conducted in collaboration with the National Kala-azar Elimination Programme (NKEP) and under the guidance of local UHC. Trained field research assistants (FRAs) visited households in the study areas with designated health inspectors and assistant health inspectors. They asked the household head(s) for any family member with VL/ PKDL at any time in the past and household member currently with PKDL-like skin lesions. If the answer was no, they skipped the household and went for the next family. The field workers cross-checked the negative answers using the VL patient database at the UHCs. In case any household head replied yes, the research team completed consenting procedures first and then screened household member(s) for PKDL-like lesions with or without a past history of VL. In case the person having a lesion was absent at that time, the research team revisited the household for testing and further procedures. Upon acknowledging having a PKDL-like skin lesion member in the house, none of the household heads refused to participate in the screening and subsequent activities. FRAs conducted an rK39 rapid test using finger prick blood among household member(s) with PKDL-like skin lesions. The team referred rK39 positive case(s) to the Upazila health complex for further confirmation of PKDL diagnosis and management following national kala-azar case management guidelines [13]. In the field, household members with skin lesions but negative for the rK39 rapid test were requested to seek medical care from the Upazila Health Complex.

### Interview of PKDL patients for knowledge about VL/PKDL and their perception of stigma

The research team interviewed PKDL cases for their knowledge and practice about VL and PKDL and their perception of stigma related to their skin lesions due to PKDL. The team used a structured interview questionnaire to assess patients’ knowledge and practice of VL/PKDL. They also used a 12-item semi-structured explanatory model interview catalog (EMIC) stigma scale to score PKDL patients’ perceptions of stigma. The scale is widely used in other diseases with skin lesions [14]. The EMIC comprises 12 questions that assess various feelings and experiences indicative of stigma, rated on a four-point Likert scale (“Yes” = 3; “Possibly” = 2; “Uncertain” = 1; and “No” = 0). Respondents who answer “Yes” are considered to exhibit a strong indication of stigma and receive the highest score (e.g., three points), while those responding “No” are deemed to have no stigma feelings and are assigned the lowest score (e.g., zero points). However, question number two is scored in reverse. The EMIC total score ranges from 0 to 36, with higher scores indicating greater levels of stigma.

### Lab test

Trained field research assistants conducted the rK39 rapid test using finger prick blood following the manufacturer's instructions.

### Data management and analysis

We used EPI Info Version 7 [15] for data management and IBM SPSS Statistics 25 (IBM SPSS, Chicago, IL) for data analysis. Data were checked and cleaned before analysis. The study statistician conducted mostly descriptive analysis and calculated the prevalence of PKDL following the standard formula for PKDL prevalence. The chi-square test was used to examine the association between categorical variables. We conducted binary logistic regression to determine the odds ratios (ORs) and their corresponding 95 % confidence intervals (95 % CIs) for analyzing both univariate and multivariable associations between variables.

### Ethical considerations

The study activities started after obtaining approval from the ICDDR,B Ethical Review Committee (PR-20138), and WHO Ethical Review Committee (ERC.0003529). Trained research assistants obtained informed voluntary written consent and assent (where applicable) from household heads of suspected and confirmed PKDL cases before the introduction of any study-related activities.

## Results

### Study areas, population and their demography, and VL burden

The study included 102 (13.1 %) VL endemic villages in five upazilas with 108637 households with a population of 472,435. Fulbaria upazila had the highest percentage of VL endemic villages (28 %), followed by Trishal, Bhaluka, Gafforgaon, and Madhupur. The proportion of households with reported VL cases, however, was highest in Trishal (5.9 %), followed by Madhupur, Fulbaria, Gafforgaon, and Bhaluka. In the VL endemic villages of five upazilas, about 1 % of inhabitants suffered from VL at any time in the past, which was the highest in Trishal (1.39 %) (Table 1).

**Table 1:** Characteristics of VL endemic upazilas and their VL burden.

Sub-districts			Bhaluka % (n/N)	Fulbaria % (n/N)	Gaffargaon % (n/N)	Madhupur % (n/N)	Trishal % (n/N)	Total % (n/N)
VL endemic villages in the upazila			10.9 (12/110)	28.4 (33/116)	7.9 (17/214)	6.7 (12/180)	17.7 (28/158)	13.1 (102/778)
Households with past Kala-azar in VL endemic villages			1.47 (213/14516)	2.75 (1262/45908)	2.26 (374/16535)	2.79 (118/4229)	5.09 (1397/27449)	3.10 (3364/108637)
VL-affected people in the VL endemic villages			0.43 (258/60708)	0.74 (1491/201717)	0.62 (437/70853)	0.80 (135/16956)	1.39 (1701/122201)	0.85 (4022/472435)
Household members of VL patients screened for PKDL			980	5976	1807	537	6905	16205
Suspected PKDL around a past case of VL			0.82 (8/980)	0.40 (24/5976)	0.44 (8/1807)	1.30 (7/537)	0.51 (35/6905)	0.51 (82/16205)
Confirmed PKDL around a past case of VL			0.82 (8/980)	0.30 (18/5976)	0.44 (8/1807)	0.75 (4/537)	0.35 (24/6905)	0.38 (62/16205)
Distribution of confirmed PKDL	Newly detected PKDL cases	PKDL with a past history of VL (n)	4	10	4	1	12	31
		PKDL without a past history of VL (n)	1	1	0	0	2	4
	Past PKDL cases	PKDL with a past history of VL (n)	3	7	4	3	9	26
		PKDL without a past history of VL (n)	0	0	0	0	1	1
Prevalence of PKDL (all confirmed PKDL/Population) x 10000			1.3	0.9	1.1	2.4	2.0	1.3

### PKDL burden

The household members of 4022 individuals with a history of VL were screened for PKDL. Among 16,205 people screened, 82 cases (0.51 %) were suspected of PKDL. Medical officers in UHCs confirmed PKDL in 62 of 82 (76 %) suspected cases. Thirty-five of the confirmed cases were new PKDL cases, and twenty-seven cases were treated at any time in the past with no improvement (defined herein as past PKDL). Four of 35 new PKDL cases and 1 of 27 past PKDL cases had no history of VL. New and past PKDL cases in the study areas made a PKDL prevalence of 0.013 % (1.3 (95 % CI: 1.0-1.7) in 10,000 people in the endemic villages). The distribution of PKDL prevalence showed that it was highest in Madhupur (0.024 %) upazila and lowest in Fulbaria (0.009 %) upazila (Table 1).

### Characteristics of confirmed PKDL cases

Among 16,205 people screened for PKDL, almost half of them (48 %) were 15-43 years old. The majority of the confirmed PKDL patients (53 %) were also from the same age group. Most of the confirmed cases were illiterate (55 %), farmers (37 %) and married (85 %). Males were predominant among the confirmed PKDL (65 % vs. 35 %) and population screened (53 % vs 47 %) in the study. The difference in age and gender among PKDL and non-PKDL populations was not statistically significant (Table 2 and Supplementary Material 5).

**Table 2:** Characteristics of 62 PKDL patients.

Characteristics	PKDL (N = 62) % (n)
**Age distribution (in years)**	
<15	8 (5)
15-43	53 (33)
≥44	39 (24)
**Gender distribution**	
Male	65 (40)
Female	35 (22)
**Marital status**	
Married	85 (53)
Unmarried	15 (9)
**Educational status**	
Illiterate	55 (34)
Informal education	10 (6)
Primary education	14 (9)
Secondary education	15 (9)
Higher education & above	6 (4)
**Occupation**	
Farmer	37 (23)
Housewife	24 (15)
Business	5 (3)
Student	12 (7)
Labor	15 (9)
Unemployed	1 (1)
Job in office	7 (4)

### Healthcare-seeking behavior of confirmed PKDL patients

We interviewed 62 confirmed cases of PKDL for their healthcare-seeking behavior. Less than half of the cases (27/62) confirmed that they sought healthcare for their skin lesions after the onset of skin lesions, as 59 % (16/27), 37 % (10/27), and 4 % (1/27), respectively, went to a qualified doctor in the public hospitals, local informal health care providers and qualified private doctors for their first consultation. Finally, qualified doctors in the public hospitals made their diagnosis, and 27 of 62 patients received treatment for PKDL. Most of the cases had macular lesions (97 %). Cases with lesions in both the exposed and unexposed body parts were more common (58 %), followed by cases with lesions in only exposed (31 %) and unexposed (11 %) body parts. Sixteen of 27 (59 %), 10/27 (37 %), and 1/27 (4 %) received, respectively, Miltefosine monotherapy, Ambisome monotherapy, and their combination for treatment. Twenty-two percent (6/27) had an interruption of treatment due to side effects, mostly with Miltefosine. The unavailability of drugs and migration were the other reasons for treatment interruption (Table 3).

**Table 3:** Healthcare-seeking behavior and treatment of confirmed PKDL patients.

Indicators (N = 62)	% (n)
**Seek health care for skin lesions**	
Yes	44 (27)
No	56 (35)
**Go for the first consultation**	
Local informal healthcare provider	37 (10)
Private qualified doctor	3 (1)
Government doctor	60 (16)
**Lesions location**	
Exposed parts	11 (7)
Unexposed parts	31 (19)
Both	58 (36)
**Type of skin lesions**	
Macular	96 (60)
Papular	2 (1)
Nodular	2 (1)
Polymorphic	0 (0)
**Treatment for PKDL**	
Miltefosine	59 (16)
Ambisome	37 (10)
Combination of drugs	4 (1)
**Interruption of treatment**	
Yes	22 (6)
No	78 (21)
**Duration of treatment interruption (in weeks)**	
<1 week	17 (1)
>3 week	83 (5)
**Reason for interrupted treatment**	
Side effects	50 (3)
No drugs available	33 (2)
Migration	17 (1)
**Side effects**	
Miltefosine	100 (3)

Table 4 provides a comparison of various indicators between PKDL patients who sought medical attention (n = 27) and those who didn't (n = 35). The majority of the patients (71 %) who sought healthcare had lesions in the exposed parts of their bodies. Gender distribution shows a higher proportion of males in both groups. Age distribution reveals that 59.3 % of the patients aged 15-43 years sought healthcare, whereas 48.6 % of the same group didn't seek any healthcare for their skin conditions. None of the above-mentioned associations were statistically significant. We conducted univariate and multivariate logistic regression to analyze the relationship between healthcare-seeking behavior and PKDL lesion area, age, and gender. No statistically significant relationships were identified (Supplementary Material 6).

**Table 4:** Comparison of healthcare-seeking behavior among PKDL patients.

Indicators	Sought healthcare		*p*-value	Total % (n), N = 62
	Yes (N = 27) % (n)	No (N = 35) % (n)		
**PKDL lesion area**				
Exposed parts	70.4 (19)	68.6 (24)	0.879	69.4 (43)
Unexposed parts	29.6 (8)	31.4 (11)		30.6 (19)
**Gender distribution**				
Male	59.3 (16)	68.6 (24)	0.447	64.5 (40)
Female	40.7 (11)	31.4 (11)		35.5 (22)
**Age distribution (in years)**				
<15	7.4 (2)	8.5 (3)	0.701	8.1 (5)
15-43	59.3 (16)	48.6 (17)		53.2 (33)
≥44	33.3 (9)	42.9 (15)		38.7 (24)

### Patients’ knowledge about PKDL and their perceived stigma

Among the 62 PKDL cases, 57 had previous VL cases in the household. All PKDL cases had heard of VL, but only 35 (56 %) had heard about PKDL. Thirty-three PKDL cases (53 %) mentioned skin lesions as a symptom of PKDL, whereas five PKDL cases (8%) mentioned fever. Fifteen PKDL cases knew that PKDL can serve as a reservoir of VL (24 %).

Out of 36 maximal points in the stigma score, the mean score among the cases was 4.2±4.3 (min=0, max=18), implying a low level of stigma. Fifty-six participants (90.3 %) had a stigma score of less than 10, whereas twenty-three (37.1 %) had no score on the stigma scale. Only six (9.7 %) PKDL patients had a stigma score of between 10 and 20. No participants had a stigma score above 20 (Supplementary Material 1). Regarding these problematic items, item 9 had the highest proportion of 0 responses (98.4 %), followed by item 10 (91.9 %) and item 11 (90.3 %). Item 9 had only “No” and “Yes” answers from the 62 participants. Participants didn't answer “Yes” as a response to four items (8, 9, 10, 12) (Supplementary Material 2).

### Detection of PKDL cases in the leprosy facilities

Seven leprosy facilities (two leprosy hospitals and five leprosy screening centers) applied an integrated approach for detecting PKDL cases among individuals attending those facilities with skin lesions but negative for leprosy after a leprosy-specific examination. During the study period, among the attendees with skin lesions in those facilities, 323 were found negative for leprosy and were eligible for screening with rK39 rapid test for PKDL. Only 137 of 323 (42 %) consented to be tested PKDL, and we performed rK39 testing on them. Ten of 137 (7.3 %) tested positive by rK39 tests, and the diagnosis of PKDL was confirmed in all by VL expert physicians in the SKKRC (Supplementary Material 3). Six out of these ten cases were from the five high-VL endemic upazillas. However, they were from villages that didn't report VL cases between 2018-2021, so they were not actively searched and without any chance of double counting.

## Discussion

This is the first study to explore the PKDL burden in the high VL endemic upazilas in Bangladesh after achieving the VL elimination target. The study found that the prevalence of PKDL in Bangladesh is 1.3 per 10,000 population (62/472435) within the VL endemic villages of the high VL endemic upazilas with VL case reports for the period 2018-2021. Six out of 10 cases detected through leprosy facilities were from six villages of the study upazilas, but these villages did not report VL cases for the period 2018-2021 and did not meet the criteria for our study villages. Adding those six cases to 62 PKDL cases would give an estimated PKDL prevalence of 1.4 per 10,000 people in a village in the high-endemic upazilas. The net increment is about 5 % (considering the actual number of PKDL prevalence, not the rounded one) in PKDL prevalence, indicating that most PKDL yield will be derived from ACD for PKDL in the villages with recently reported cases of VL. However, the best scenario would be ACDs in all villages with cases of VL at any time in the past, which might be difficult from the program's perspective. Our study showed that a house-to-house survey in the villages with recent reports of VL would result in a substantial yield of PKDL case detection complemented by ACDs involving other facilities like leprosy hospital(s) and screening center(s).

The existence of PKDL cases is a concern, though its burden is now far less compared to that during the attack phase of the VL elimination program [5]. A survey done in the Mymensingh district in 2008 found a PKDL prevalence of 6.2 per 10,000 population; however, they used a different methodology [16]. A population-based study done in Fulbaria, Mymensingh, Bangladesh, during the attack phase detected 79 (9.7 %) PKDL cases among the participants searched [5]. Although the active case search method was similar to ours, the objectives were pretty different. A study done in Nepal following a similar methodology found a PKDL prevalence of 2.23 per 10,000 population [17]. The lower PKDL prevalence in our study reflects the success of the NKEP in bringing down the VL burden to less than 1 per 10,000 people in all VL endemic upazilas of the country, as PKDL is a sequel of VL in most of the cases. Further, Bangladesh is the first country globally to eliminate VL as a public health problem. The study result establishes what the burden of PKDL could be in this situation, which may serve as an example for other VL-endemic countries, targeting VL elimination as a public health problem. Further, the WHO NTD roadmap targets a decrease of PKDL burden by 70 % and 100 %, respectively, in 2006 and 2030. But currently, there is no baseline. Our study result can be taken by the NKEP to fill this gap. The study also demonstrated that the well-developed hierarchal infrastructure of the public health system in Bangladesh successfully facilitated an investigation/ survey like this. The NKEP should continue to do that to achieve its final destination of zero transmission of VL by 2030 by bringing all PKDL cases under treatment. The surveillance can be further strengthened if all community clinics in VL endemic areas can be added to active VL and PKDL searches. Each community clinic provides primary health care for six thousand people.

The clinical presentation, age, and sex distribution of the cases remained the same as before, as a macular form of the disease is more frequent [16,18,19], the male sex predominates over the female sex [20,21], and the condition is more common among individuals over 15 years old [16,18,20]. Regarding the distribution of PKDL by sex, our study differed from that of Ghosh P et al., where female predominance was observed [22]. However, males are more susceptible to intracellular pathogens such as leishmaniasis, tuberculosis, lepromatous leprosy, salmonellosis, leptospirosis, and hepatitis A due to sex hormones, sex-specific behavior, increased exposure risk, and higher treatment-seeking behavior [[23], [24], [25]]. In contrast, females have a better immune response due to the higher number of immune-related genes on the X chromosome and the dosage effects of this chromosome [24,26]. A study conducted in India established a male predominance in PKDL, showing a strong association with testosterone [27]. The majority of the PKDL patients found in this study were from the 15-43 years age group. This coincides with Mukhopadhyay et al., who also concluded that PKDL mainly occurs in the postpubertal age group, indicating that sex hormones may play a role [27,28]. Several recent studies revealed that the knowledge, attitude, and practice of the community about PKDL remained unsatisfactory in India and Nepal [17,28]. In our case, it was a bit better as we found that there are some improvements regarding PKDL awareness and healthcare-seeking behavior of the affected individuals, as about 56 % (35/62) and 43 % (27/62) sought knowledge about PKDL and healthcare for their skin condition, respectively. These proportions are higher than those reported by us and others from Bangladesh in the past [29]. These indicate that awareness campaigns by the national program, researchers, and other agencies on VL and PKDL might have impacted people's knowledge about VL and PKDL. The national program should continue these activities during the next phase of the program.

Despite some improvement in the healthcare-seeking behavior of PKDL cases, as mentioned above, it still remains below 50 %. Therefore, active case search for PKDL cases to bring them under management to prevent transmission of VL is of utmost importance. A study in India found a clustering of cases of leprosy and PKDL in skin camps for PKDL [30]. This is explainable as the clinical presentation of both diseases is very similar. Therefore, we thought of an active search for PKDL cases in leprosy facilities among leprosy test-negative individuals. Though a similar approach in Nepal gave frustrating results [17], we found the approach very fruitful. We found 10 cases through screening of 137 leprosy-negative leprosy suspects in the two leprosy hospitals and in five leprosy screening centers. It makes a significant yield of 7 % (10/137) PKDL in addition to cases with minimum investment for training in conducting rK39 rapid test by the health workers/lab technicians in the leprosy hospitals and cost for the rK39 test, which is less than 2 USD per test. Implementing PKDL detection activity into existing leprosy control programs can leverage the limited resources we have, leading to more efficient and cost-effective disease surveillance. Combining control efforts for both diseases can lead to synergies in surveillance, diagnosis, treatment, and community engagement, maximizing the impact of interventions.

Currently, oral Miltefosine is the only option for the treatment of PKDL in the Indian sub-continent. Patients must be treated with hundred percent compliance to avoid the emergence of drug resistance and eventual relapse of PKDL after the cure. The present study found that PKDL patients experienced treatment interruption due to the unavailability of medicines, which is a big concern and, if it continues, will be a significant threat to the success of the VL elimination program. The present study demonstrated that about 22 percent of PKDL cases could not complete their treatment due to the unavailability of medicine. We hope that this finding will sensitize the program to fill up this gap in the best way.

Similar to leprosy, PKDL patients may face stigma, which affects their treatment-seeking behavior. Although in the present study, the mean stigma score among PKDL patients was very low, some of them had their stigma level as high as was observed in a study in Bihar, India [31]. A study in Brazil among leprosy patients found an EMIC stigma score as high as 18.8 [32]. On the other hand, in spite of having a very low stigma associated with PKDL, less than half of the patients sought medical attention, indicating potential barriers to access or awareness regarding available healthcare services, cultural beliefs, or logistical challenges in accessing medical care. Awareness programs on VL and PKDL and health education on PKDL during treatment of VL should help these people seek health care at an early stage of the disease so that they can prevent stigmatization. All these activities are necessary to sustain the VL elimination success achieved in recent times.

The study has some limitations. We asked the household heads regarding the presence of any PKDL-like skin lesion cases in the house. There was some chance of underreporting of cases due to recall bias and stigma. However, we involved local government field staff working in the locality in VL elimination for years in the house-to-house survey. Moreover, we used historical VL data kept in the concerning UHC to mitigate this concern of underreporting. Further, we could test 48 % of leprosy-negative cases for PKDL, which also contributed to underreporting up to some extent. It was surprising that 58 % of leprosy-negative cases did not consent to testing for PKDL. We did not explore the reason behind this, but better motivation and medical education to improve this situation are highly needed. We did not do parasitological confirmation of probable PKDL cases reported in this study. This is because it does not add additional value from program perspectives as both probable and confirmed cases of PKDL are eligible for treatment as per national guidelines for kala-azar and PKDL case management. Further, lack of a gold standard laboratory diagnostic tool for PKDL, as microscopic examination of slit skin aspirate or skin tissue specimens has poor sensitivity of diagnosis of PKDL, and a molecular tool like qPCR has isolation rate of LD DNA from skin biopsy is about 80 % in particular in macular cases of PKDL which is the most common form of PKDL in Bangladesh. qPCR needs highly trained personnel and expensive equipment and cannot serve as a point of care/need test. Its high cost is also a hurdle from a program perspective. Another limitation is that we could not address atypical cases of PKDL in the community [[24], [25], [26]]. However, only one report of atypical PKDL from Bangladesh in the last two decades might indicate that its burden may not be high [33]. But it is important to find them and bring them under treatment. Since most of these dermatological cases seek medical care from dermatologists, their sensitization about active research for PKDL will be worth it [20]. The National Program may undertake activities to sensitize all dermatologists in the country on PKDL case detection and management. Another limitation was that we couldn't compare the values of this study with those of other similar studies due to the differences in methodologies.

## Conclusions

PKDL exists in the VL endemic villages of Bangladesh, though its burden is now less than that in the past [16]. Integration of leprosy hospitals and screening centers in active search for PKDL will be worth it as this will further strengthen the National Program strategy in PKDL case detection. PKDL treatment interruption is a concern and should be solved as soon as possible. The program should continue its awareness campaigns as it helps raise people's awareness about PKDL and can prevent them from stigma. Finally, the NKEP may consider study results as a baseline for PKDL burden in VL endemic villages of highly VL endemic upazilas to achieve the target of the WHO roadmap to bring down PKDL burden by 70 % and 100 % in 2026 and 2030, respectively.

## Availability of data and materials

The data underlying the results presented in this study will be provided by Dr. Dinesh Mondal at a reasonable request. Email: din63d@icddrb.org.

## Declaration of competing interest

The authors have no conflicts of interest associated with the material presented in this paper.

## Appendix Supplementary materials

Image, application 1

Image, application 2

Image, application 3

Image, application 4

Image, application 5

Image, application 6
