# Supplementary material for: Post kala-azar dermal leishmaniasis burden at the village level in selected high visceral leishmaniasis endemic upazilas in Bangladesh
Source: Int J Infect Dis. 2024 Oct;147:None. doi: 10.1016/j.ijid.2024.107213 (PMC11442318; doi:10.1016/j.ijid.2024.107213)
Supplement: Supplementary file 1 [file mmc1.docx]

**Table: Stigma score of 62 PKDL patients**

| **Particulars** | **Values** |
| --- | --- |
| Mean (±SD) stigma score | 4.2±4.3 (min=0, max=18) |
| **Frequency of stigma score** | |
| <10 | 90 (56) |
| 10-20 | 10 (6) |
| 21-30 | 0.0 (0) |
| >30 | 0.0 (0) |
