# Supplementary material for: Post kala-azar dermal leishmaniasis burden at the village level in selected high visceral leishmaniasis endemic upazilas in Bangladesh
Source: Int J Infect Dis. 2024 Oct;147:None. doi: 10.1016/j.ijid.2024.107213 (PMC11442318; doi:10.1016/j.ijid.2024.107213)
Supplement: Supplementary file 2 [file mmc2.docx]

Fig 1: Answer profile to each item of the Explanatory Model Interview Catalog -Stigma Scale by 62 persons affected by PKDL

**Figure: Answer profile to each item of the Explanatory Model Interview Catalog -Stigma Scale by 62 persons affected by PKDL**

The twelve items in the EMIC scale include:

Item 1: Have you tried to keep your condition a secret and keep others away from knowing?

Item 2: Have you felt ashamed or embarrassed or think less of yourself because of your skin condition?

Item 3: Do you feel that people have insulted or made fun of you because of your condition?

Item 4: Do you think people have shown a lack of respect for you because of your condition?

Item 5: Do you think people have considered that your skin condition might have an adverse effect on others?

Item 6: Do you think other people have avoided you because of your condition?

Item 7: Do you think people have avoided visiting your house because of your condition?

Item 8: Do you think people have thought less about your family due to your skin condition?

Item 9: Have you felt that your condition has caused problems with your family life (if unmarried)?

Item 10: Have you felt that your condition has caused problems with your marriage prospects (if married)?

Item 11: Have you been asked to stay away from work due to your skin condition?

Item 12: Have you experienced problems with making friends or maintaining friendships because of your condition?
