# Supplementary material for: Post kala-azar dermal leishmaniasis burden at the village level in selected high visceral leishmaniasis endemic upazilas in Bangladesh
Source: Int J Infect Dis. 2024 Oct;147:None. doi: 10.1016/j.ijid.2024.107213 (PMC11442318; doi:10.1016/j.ijid.2024.107213)
Supplement: Supplementary file 3 [file mmc3.docx]

**Table: Yields of integration of Leprosy hospitals in endemic areas for active detection of PKDL cases**

| Sub-districts | Name of Leprosy facilities | Time duration | Total Leprosy Positive | Total leprosy negative | rK39 test done  % (n) | rK39 positive  % (n) |
| --- | --- | --- | --- | --- | --- | --- |
| Bhaluka | Damien Foundation Leprosy Screening Center, Bhaluka | July 2021 to June 2022 | 00 | 40 | 53 (21) | 0.0 (0) |
| Fulbaria | Damien Foundation Leprosy Screening Center, Fulbaria | July 2021 to June 2022 | 02 | 57 | 30 (17) | 0.0 (0) |
| Gaffargaon | Damien Foundation Leprosy Screening Center, Gaffargaon | July 2021 to June 2022 | 01 | 63 | 33 (21) | 0.0 (0) |
| Madhupur | Damien Foundation Leprosy Screening Center, Madhupur | July 2021 to June 2022 | 00 | 18 | 28 (5) | 0.0 (0) |
|  | Jolchotro Leprosy Hospital | July 2021 to June 2022 | 31 | 62 | 79 (49) | 16 (8) |
| Trishal | Damien Foundation Leprosy Screening Center, Trishal | July 2021 to June 2022 | 00 | 75 | 28 (21) | 5 (1) |
| Mymensingh  Sadar | Shambhugonj Leprosy Hospital | July 2021 to June 2022 | 16 | 8 | 38 (3) | 33 (1) |
| Total | | July 2021 to June 2022 | 50 | 323 | 42.4 (137) | 7.3 (10) |
