# Supplementary material for: Post kala-azar dermal leishmaniasis burden at the village level in selected high visceral leishmaniasis endemic upazilas in Bangladesh
Source: Int J Infect Dis. 2024 Oct;147:None. doi: 10.1016/j.ijid.2024.107213 (PMC11442318; doi:10.1016/j.ijid.2024.107213)
Supplement: Supplementary file 4 [file mmc4.docx]

**Table: EMIC Stigma Scale**

| **Items** | **No** | **Uncertain** | **Possibly** | **Yes** |
| --- | --- | --- | --- | --- |
| 1. Keep others from knowing |  |  |  |  |
| 1. Think less of yourself |  |  |  |  |
| 1. Embarrassed or shamed |  |  |  |  |
| 1. Receive less respect from other |  |  |  |  |
| 1. Adverse effect on others |  |  |  |  |
| 1. Others avoided you |  |  |  |  |
| 1. Refuse to visit your home |  |  |  |  |
| 1. Others think less of your family |  |  |  |  |
| 1. Difficult to marry (if unmarried) |  |  |  |  |
| 1. Problems in family (if married) |  |  |  |  |
| 1. Asked to stay away from work |  |  |  |  |
| 1. Decided to stay away from social groups |  |  |  |  |
