# Supplementary material for: Post kala-azar dermal leishmaniasis burden at the village level in selected high visceral leishmaniasis endemic upazilas in Bangladesh
Source: Int J Infect Dis. 2024 Oct;147:None. doi: 10.1016/j.ijid.2024.107213 (PMC11442318; doi:10.1016/j.ijid.2024.107213)
Supplement: Supplementary file 5 [file mmc5.docx]

**Table: Age and gender distribution among PKDL patients and screened populations**

| **Characteristics** | **PKDL (n=62)**  **% (n)** | **Total (n=16205)**  **% (n)** |
| --- | --- | --- |
| **Age distribution (in years)** | | |
| <15 | 8.1 (5) | 28.4 (4598) |
| 15-43 | 53.2 (33) | 47.7 (7730) |
| ≥44 | 38.7 (24) | 23.9 (3877) |
| **Gender distribution** | | |
| Male | 64.5 (40) | 52.5 (8506) |
| Female | 35.5 (22) | 47.5 (7699) |
