# Supplementary material for: Post kala-azar dermal leishmaniasis burden at the village level in selected high visceral leishmaniasis endemic upazilas in Bangladesh
Source: Int J Infect Dis. 2024 Oct;147:None. doi: 10.1016/j.ijid.2024.107213 (PMC11442318; doi:10.1016/j.ijid.2024.107213)
Supplement: Supplementary file 6 [file mmc6.docx]

**Table: Univariate and Multivariate logistic regression analyses between healthcare-seeking behavior and other variables**

| **Health care** | **Unadjusted**  **OR (95% CI)** | **P value** | **Adjusted**  **OR (95% CI)** | **P value** |
| --- | --- | --- | --- | --- |
| **Lesion location/Area** |  |  |  |  |
| Exposed parts | Reference |  | Reference |  |
| Unexposed parts | 0.92 (0.31, 2.74) | 0.879 | 0.81 (0.26, 2.52) | 0.720 |
| **Gender distribution** |  |  |  |  |
| Male | Reference |  | Reference |  |
| Female | 1.50 (0.53, 4.28) | 0.448 | 1.49 (0.47, 4.66) | 0.496 |
| **Age distribution (in years)** |  |  |  |  |
| <15 | Reference |  | Reference |  |
| 15-43 | 1.41 (0.21, 9.58) | 0.724 | 1.63 (0.23, 11.68) | 0.627 |
| ≥44 | 0.90 (0.13, 6.46) | 0.917 | 1.11 (0.14, 9.14) | 0.922 |
